# The predictive value of crescents in the disease progression of lupus nephritis based on the 2018 International Society of Nephrology/Renal Pathology Society Revision System: a large cohort study from China

**DOI:** 10.1080/0886022X.2020.1726385

**Published:** 2020-02-13

**Authors:** Juan Tao, Hui Wang, Su-Xia Wang, Feng Yu, Ming-Hui Zhao

**Affiliations:** a Renal Division, Department of Medicine, Peking University First Hospital, Beijing, People’s Republic of China; b Institute of Nephrology, Peking University, Beijing, People’s Republic of China; c Key Laboratory of Renal Disease, Ministry of Health of China, Beijing, People’s Republic of China; d Key Laboratory of CKD Prevention and Treatment, Ministry of Education of China, Beijing, People’s Republic of China; e Laboratory of Electron Microscopy, Peking University First Hospital, Beijing, People’s Republic of China; f Department of Nephrology, Peking University International Hospital, Beijing, People’s Republic of China; g Peking-Tsinghua Center for Life Sciences, Beijing, People’s Republic of China

**Keywords:** Crescents, lupus nephritis, prognosis, renal biopsy

## Abstract

**Objective:**

This study analyzed the associations of different crescents’ fraction and clinical features in a Chinese lupus nephritis cohort based on the 2018 revision of ISN/RPS classification system.

**Methods:**

A total of 288 lupus nephritis patients with complete clinicopathological data and well follow-up was enrolled. The fraction of glomeruli with cellular or fibrocellular crescents based on the new system was reevaluated. The association between crescents fractions and the outcomes were further analyzed.

**Results:**

The median follow-up period was 76.5 months. Cellular or fibrocellular crescents were present in 146 (50.7%) of the total individuals. The percentage of crescents were significantly associated with severe clinical renal injury indices and other renal pathological parameter. According to the survival receiver operating characteristic (survival ROC) curve, the optimal cutoff level of cellular or fibrocellular crescents for predicting the composite events was 7.39%. By multivariable Cox hazard analysis, the presence of crescents was predictive of survival from the composite events with a hazard ratio [HR] of 2.5 (95% CI 1.190–5.431; *p* = .02). Furthermore, when we used absent, present in less than 7.39% of glomeruli, and present in greater than or equal to 7.39% of glomeruli as cutoffs in all the patients, a gradation appeared, with adjusted HRs of 2.9 (95% CI 1.326–6.313; *p* = .008) for crescents in greater than or equal to 7.39% of glomeruli, in reference to no crescents.

**Conclusion:**

We proposed that the crescents were not uncommon and had important clinical significance in lupus nephritis. The cutoff point of crescents as prognosticator might be nearly 7.39%.

## Introduction

Renal involvement is common in systemic lupus erythematosus (SLE) and the pathological phenotypes of lupus nephritis varied a lot. Proliferation, a key feature of active lupus nephritis, includes endocapillary hypercellularity and extracapillary hypercellularity (crescents). Crescents are fairly not uncommon in biopsy samples from lupus nephritis patients [1,2]. Our previous study suggested that the patients with “true” crescentic glomerulonephritis presented with more severe outcomes in lupus nephritis [3], which was consistent with other reports [1,4,5], although the effect of different fractions of crescents was not determined yet. Importantly, a 2018 revision of the International Society of Nephrology/Renal Pathology Society (ISN/RPS) lupus nephritis classification system has been established recently, in which more clear definitions are provided for cellular, fibrocellular and fibrous crescents [6].

Herein, the pathological active (cellular or fibrocellular) crescents were reevaluated, and their clinical significances were further analyzed in a large well-defined lupus nephritis cohort based on the 2018 system.

## Materials and methods

### Patients

Two hundred eighty-eight patients who underwent renal-biopsy from April 1994 to September 2017, with biopsy-proven lupus nephritis in Peking University First Hospital, were collected. Patients with ≥10 scorable glomeruli and fulfilling ≥4 of the 1997 American College of Rheumatology revised criteria for SLE [7] were included. All patients’ data were coded and kept anonymous.

### Clinical and laboratory data

The demographic data collected included age and gender at the time of biopsy. Clinical parameters were within 3 months of date of biopsy. The clinical disease activity was assessed by the Systemic Lupus Erythematosus Disease Activity Index (SLEDAI) [8,9]. Serum antinuclear antibodies and anti-double-stranded DNA were detected using indirect and Crithidia luciliae indirect immunofluorescence assay respectively (EUROIMMUN, Lübeck, Germany). Serum C3 was determined using rate nephelometry assay (Beckman-Coulter, IMMAGE, USA, normal range >0.85 g/L).

Follow-up was scheduled every 1–6 months depending on the patient’s status or the treating physician. The primary event of interest was a combined event defined by ≥30% reduction from baseline estimated glomerular filtration rate (eGFR), end-stage renal disease (ESRD; eGFR <15 mL/min/1.73m^2^, requirement for maintenance dialysis for at least 6 months, or kidney transplantation), or death. Patients lost to follow-up or those who did not reach the event of interest during follow-up were censored. The outcome was time to the earliest of the composite renal event and the censoring event. Survival time was defined from baseline (kidney biopsy) until the date of the last follow-up, the incidence of the event of interest, or December 31, 2018 (end of the study).

Informed consent was obtained for renal biopsy from each patient. The research was in compliance with the Declaration of Helsinki. The design of this work was approved by the local ethical committees [Peking University First Hospital, approval number: 2017(1333)].

### Renal histopathology

Renal biopsy specimens were fixed in 4% buffered formaldehyde for light microscopy. Consecutive serial 3 µm sections were used for histological staining, including hematoxylin and eosin, periodic acid–silver methenamine and Masson’s trichrome. Slides were reviewed by two experienced nephropathologists. Differences in scoring between the two pathologists were resolved by re-reviewing the biopsies and coming to a consensus.

For each biopsy, we determined the fraction of glomeruli with cellular or fibrocellular crescents based on the 2018 ISN/RPS revised lupus nephritis classification system [6]. A crescent was defined as a lesion consisting of extracapillary hypercellularity, composed of a variable mixture of cells. Fibrin and fibrous matrix may be present; 10% or more of the circumference of Bowman’s capsule should be involved. A cellular crescent was defined by more than 75% cells and fibrin and less than 25% fibrous matrix, and a fibrocellular crescent was defined by 25–75% cells and fibrin and the remainder fibrous matrix (Supplementary Figure 1). Fibrous crescents (composed of more than 75% fibrous matrix and less than 25% cells and fibrin) were not taken into account. However, in the 2003 ISN/RPS pathologic classification of lupus nephritis [10], extracapillary hypercellularity involving >25% of the circumference of Bowman’s capsule was original cutoff, and there were no definitions for fibrous or fibrocellular crescents.

### Statistical analysis

Normally distributed variables were expressed as mean ± SD. Nonparametric variables were expressed as median and interquartile range and compared using either Mann–Whitney or Kruskal–Wallis. Categorical variables were expressed in percentages. Correlations between crescents and clinical features were carried out using the Spearman’s test. The survival receiver operating characteristic (survival ROC) curve analysis was used to evaluated the prognostic value of crescents for the composite endpoint. The maximum value of the area under the ROC curve (AUC) was used as a criterion for selecting the optimum cutoff point. The composite outcome was used as the status variable. Kaplan–Meier curves were used to analyze patients’ prognosis. Survival analysis using univariate and multivariable Cox regression was performed to test the association between crescents and a composite event (≥30% reduction from baseline of eGFR, ESRD or death, to increase the rate of events and permit a valid multivariable analysis). The following variables were assessed as the covariates adjusted: age, gender, proteinuria, eGFR, and NIH activity/chronicity index at biopsy.

All *p* values were two tailed, and values less than .05 were considered statistically significant. Confidence intervals included 95% of predicted values. Analysis was performed with SPSS statistical software package (SPSS V.24.0; IBM) and R-software.

## Results

### Cohort description

Clinical characteristics of 288 lupus nephritis patients at the time of initial renal biopsy and during follow-up were shown in Table 1. The median age was 30.5 years, with female predominance (84.4%).

**Table 1.** Clinical characteristics of the lupus nephritis patients at the time of biopsy and follow-up.

At time of biopsy		Follow-up	
Number of patients	288	Duration of follow-up (median and interquartile range) (m)	76.5 (44.0–123.0)
Age (median and interquartile range) (years)	30.5 (23–41)	Death (%)/follow-up time	11 (3.8)/64.0 (12.0–101.0)
Female (%)	243 (84.4)	End-stage renal disease (%)/follow-up time	18 (6.3)/35.0 (12.0–142.8)
Number of hypertension (BP ≥ 140/90 mm Hg) (%)	153 (53.1)	≥30% reduction from baseline of eGFR (%)/follow-up time	30 (10.4)/92.5 (61.3–130.0)
Number with nephrotic syndrome (%)	176 (61.1)	Composite events (%)/follow-up time	59 (20.5)/82.0 (26.0–125.0)
Number with leukocyturia (noninfection) (%)	162 (56.3)		
Number with hematuria (%)	224 (77.8)		
Number with acute kidney injury (%)	66 (22.9)		
Number with anemia (%)	154 (53.5)		
Number with leukocytopenia (%)	85 (29.5)		
Number with thrombocytopenia (%)	58 (20.1)		
Number with neurological disorder (%)	13 (4.5)		
SLEDAI (mean ± SD)	17 ± 6		
Hemoglobin (mean ± SD) (g/L)	106.2 ± 23.1		
Serum creatinine value (median and interquartile range) (umol/L)	77.0 (61.0–117.0)		
Urine protein amount (median and interquartile range) (g/24 h)	3.9 (1.7–6.3)		
C3 level (median and interquartile range) (g/L)	0.42 (0.33–0.59)		
Number with low C3 level (%)	266 (92.4)		
Number with positive ANA (%)	278 (96.5)		
Number with positive anti-dsDNA antibodies (%)	185 (64.2)		
Histologic classes, *n* (%)			
I	0 (0)		
II	8 (2.8)		
III (III/III + V)	59 (20.5)		
IV (IV/IV + V)	167 (58.0)		
V	54 (18.8)		
VI	0 (0)		
Induction immunosuppressive therapy, *n* (%)			
No immunosuppressants	7 (2.4)		
CS alone	14 (4.9)		
CS + CYC	197 (68.4)		
CS + MMF	39 (13.5)		
CS + AZA	4 (1.4)		
CS + LMF	14 (4.9)		
CS + CsA	13 (4.5)		

*Note*: m: months; BP: blood pressure; SLEDAI: systemic lupus erythematosus disease activity index; ANA: antinuclear antibodies; dsDNA: double-stranded DNA; eGFR: estimated glomerular filtration rate; CS: corticosteroids; CYC: cyclophosphamide; MMF: mycophenolate mofetil; AZA: azathioprine; LMF: leflunomide; CsA: cyclosporin.

The median follow-up time was 76.5 months (interquartile range: 44.0–123.0 months). During the follow-up period, 11 patients died and 18 patients progressed to ESRD, while 30 patients with ≥30% reduction from baseline of eGFR.

### Renal biopsy findings

There was a median number of 29 glomeruli per biopsy (interquartile range: 21–37). Cellular or fibrocellular crescents were present in 146 (50.7%) patients of the total individuals, whereas 9.4% (27/288) had crescents greater than 50% of glomeruli. The distribution of the percentage of crescents observed in each biopsy is shown in Supplementary Figure 2. The median and the interquartile range of percentage of glomeruli with cellular or fibrocellular crescents in affected patients was 21.8% (9.0–37.6%).

### Associations between crescents and clinicopathological characteristics at renal biopsy

The correlations between crescents and clinical and pathological parameter at the time of renal biopsy are shown in Supplementary Table 1. There were significantly positive associations between the percentage of crescents and several clinico-pathological indices.

### Identification of the optimal categorization of the crescents fraction and adjusted predictive value of crescents

**Figure 1.**
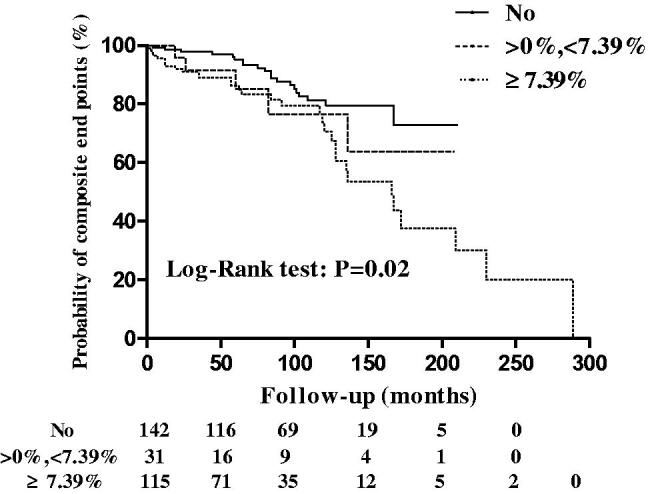
Kaplan–Meier analysis of the 7.39% of glomeruli with crescents on survival from a combined event in all patients.

The presence of the crescents was associated with a lower survival from combined events compared with its absence [hazard ratio [HR] 2.0 (95% CI 1.173–3.421; *p* = .01)]. According to the survival ROC curve, the maximum of AUC was 0.73 and the optimal cutoff level of crescents for predicting the composite events was 7.39%, whose sensitivity and specificity were 84.8% and 55.6%, respectively. Survival from the combined events declined up to greater than or equal to 7.39% of glomeruli with crescents in the total cohort [HR 2.1 (95% CI 1.221–3.689; *p* = .008)] and its Kaplan–Meier curve was shown in Figure 1. The correlations between crescents and death were shown in Supplementary Figure 3.

Multivariable analyses were performed in all the lupus nephritis patients. We considered two different categorizations of crescents as the followings: (1) present or absent only; and (2) absent, present in less than 7.39% of glomeruli, and present in 7.39% or more of glomeruli.

The presence of crescents was predictive of survival from the combined events with an HR of 2.5 (95% CI 1.190–5.431; *p* = .02) (Table 2a). When we used absent, present in less than 7.39% of glomeruli, and present in greater than or equal to 7.39% of glomeruli as cutoffs in all patients, a gradation appeared, with adjusted HRs of 2.9 (95% CI 1.326–6.313; *p* = .008) for crescents in greater than or equal to 7.39% of glomeruli, in reference to no crescents (Table 2b). Predictive values of crescents in less than 7.39% of glomeruli was not statistically significant [HR 1.6 (95% CI 0.536–4.879; *p* = 0.4)] (Table 2b).

**Table 2.** (a) Correlations between the presence of crescents and composite outcomes in all lupus nephritis patients; (b) Correlations between 7.39% of glomeruli with crescents and composite outcomes in all lupus nephritis patients.

Combined event (Cox regression)				
	Univariate hazard ratio (95% CI)	*p* Value	Multivariable hazard ratio (95% CI)*	*P* value
a. Crescents				
No	1	.01	1	.02
Any	2.0 (1.173–3.421)		2.5 (1.190–5.431)	
b. Crescents		.03		.02
No	1		1	
<7.39%	1.5 (0.568–3.990)	.4	1.6 (0.536–4.879)	.4
≥7.39%	2.1 (1.221–3.689)	.008	2.9 (1.326–6.313)	.008

*Note*: CI, confidence interval; AI, NIH activity index; CI, NIH chronicity index; eGFR, estimated glomerular filtration rate.

^*^ Multivariable: multivariable with crescents + initial age (lg transformed), gender, eGFR, proteinuria (lg transformed), AI (0–4, 5–8, >8), and CI (0–2, 3–4, 5–8). There was an increase in the hazard ratio of CI from 1 (0–2) to 4.0 (when 5–8) in Table 2a. And there was an escalation of the hazard ratio as CI increased: hazard ratio 1.9 (when 3–4) and 4.4 (when 5–8) in Table 2b. The other covariates were not statistically associated with a decreased survival from a combined event. The combined event including death, end-stage renal disease, or 30% reduction from baseline of eGFR, to increase the rate of events and permit a valid multivariable analysis, as mentioned in the text.

In order to mitigate against bias from histologic classes and treatment, two sub-group analyses were performed in patients with proliferative lupus nephritis (class III/III + V and IV/IV + V) and patients who were treated with corticosteroids plus cyclophosphamide or corticosteroids plus mycophenolate mofetil, respectively (Tables 3 and 4). Likewise, patients with crescents in greater than or equal to 7.39% of glomeruli had a significantly worse prognosis in reference to no crescents [HR 2.3 (95% CI 1.078–5.114; *p* = .03) and HR 3.5 (95% CI 1.364–9.030; *p* = .009), respectively].

**Table 3.** (a) Correlations between the presence of crescents and composite outcomes in proliferative lupus nephritis patients; (b) Correlations between 7.39% of glomeruli with crescents and composite outcomes in proliferative lupus nephritis patients.

Combined event (Cox regression)				
	Univariate hazard ratio (95% CI)	*p* Value	Multivariable hazard ratio (95% CI)*	*p* Value
a. Crescents				
No	1	0.04	1	0.07
Any	2.0 (1.029–3.735)		2.0 (0.952–4.376)	
b. Crescents		0.1		0.07
No	1		1	
<7.39%	1.5 (0.540–4.284)	0.4	1.2 (0.398–3.751)	0.7
≥7.39%	2.1 (1.067–4.000)	0.03	2.3 (1.078–5.114)	0.03

Note: CI, confidence interval; AI, NIH activity index; CI, NIH chronicity index; eGFR, estimated glomerular filtration rate.

^*^ Multivariable: multivariable with crescents + initial age (lg transformed), gender, eGFR, proteinuria (lg transformed), AI (0–4, 5–8, >8), and CI (0–2, 3–4, 5–8). There was an escalation of the hazard ratio as CI increased: hazard ratio 2.1 (when 3–4) and 5.5 (when 5–8) in Table 3a. And there was also an escalation of the hazard ratio as CI increased: hazard ratio 2.2 (when 3–4) and 6.3 (when 5–8) in Table 3b. The other covariates were not statistically associated with a decreased survival from a combined event. The combined event including death, end-stage renal disease, or 30% reduction from baseline of eGFR, to increase the rate of events and permit a valid multivariable analysis, as mentioned in the text.

**Table 4.** (a) Correlations between the presence of crescents and composite outcomes in patients who were treated with corticosteroids + cyclophosphamide/mycophenolate mofetil; (b) Correlations between 7.39% of glomeruli with crescents and composite outcomes in patients who were treated with corticosteroids + cyclophosphamide/mycophenolate mofetil.

Combined event (Cox regression)				
	Univariate hazard ratio (95% CI)	*p* Value	Multivariable hazard ratio (95% CI)*	*p* Value
a. Crescents				
No	1	0.01	1	0.02
Any	2.3 (1.216–4.344)		3.2 (1.246–8.025)	
b. Crescents		0.03		0.03
No	1		1	
<7.39%	1.6 (0.540–5.033)	0.4	2.0 (0.547–7.471)	0.3
≥7.39%	2.4 (1.275–4.705)	0.007	3.5 (1.364–9.030)	0.009

*Note*: CI: confidence interval; AI: NIH activity index; CI: NIH chronicity index; eGFR: estimated glomerular filtration rate.

^*^ Multivariable: multivariable with crescents + initial age (lg transformed), gender, eGFR, proteinuria (lg transformed), AI (0–4, 5–8, >8), and CI (0–2, 3–4, 5–8). There was an increase in the hazard ratio of CI from 1 (0–2) to 3.6 (when 5–8) in Table 4a. And there was also an increase in the hazard ratio of CI from 1 (0–2) to 4.0 (when 5–8) in Table 4b. The other covariates were not statistically associated with a decreased survival from a combined event. The combined event including death, end-stage renal disease, or 30% reduction from baseline of eGFR, to increase the rate of events and permit a valid multivariable analysis, as mentioned in the text.

## Discussion

Crescents had important clinical significance in lupus nephritis, although the effect of different fractions of crescents was not determined yet. It was showed that the cutoff level of cellular or fibrocellular crescents for predicting the composite events was 7.39%, which was fully data driven in our study.

Crescent formation is common in lupus nephritis especially in the background of proliferative glomerular lesions. In one cohort of 62 rapidly progressive glomerulonephritis (RPGN) patients with biopsy-proven crescentic glomerulonephritis, 32 patients (51.6%) had lupus nephritis [2]. Zhang et al. found that crescents accounted for 51.5% of lupus nephritis cases [1]. In our study, we also found 50.7% of lupus nephritis patients had crescents, whereas 10% had a fraction of glomeruli with one half or more crescents. It was not surprising that the percentage of crescents was significantly associated with more severe renal injury indices, like SLEDAI, serum creatinine and C3 values, proteinuria amount, and several pathological active indices in accordance with previous studies [1,3–5,11].

In our previous study, we found that the patients with “true” crescentic lupus nephritis patients (affecting ≥50% of glomeruli) had worse renal outcomes than those with pure class IV-G ones [3], which was also confirmed by other investigators later [1,4,5]. Zhang et al. [1] divided lupus nephritis patients with crescents into four subgroups according to the proportions of crescents, with <10%, 10–19%, 20–49%, ≥50%, respectively, and found that the survival was poorer along with the increase of the proportion of crescents. In the study of proliferative lupus nephritis by Cai et al. [4], patients with circumferential crescents were categorized into two groups (crescentic ratio ≤ 25%, or >25%) and >25% group had worse renal outcomes both in short and long terms. However, the optimal cutoff value of crescents’ fractions, predicting long-term outcomes precisely, has not yet been determined.

Importantly, crescent score, which including C0 (no crescents), C1 (crescents in at least 1 but <25% of glomeruli), and C2 (crescents in at least 25% glomeruli), had been added to the MEST scores of the Oxford Classification of IgA nephropathy [12], which was fully data driven and evidence based. In their previous multi-center study [13], stepwise examination of increasing fractions of crescents, from one twelfth to one fourth, were performed to determine whether the fraction of crescent-containing glomeruli associating with poor renal outcomes, and one fourth was chosen after rigorous statistical analysis. In another study by Katafuchi et al. [14], an optimal cutoff value of 7% crescents in predicting development of ESRD for IgA nephropathy were established according to the ROC curve. This highlights our further explorations on the optimal cutoff value of crescents’ fractions in lupus nephritis, especially based on the recent improved clarity of crescents’ definitions in the new version of the 2018 lupus nephritis classification system [6].

Our findings suggested that the presence of the cellular or fibrocellular crescent was identified as an independent risk factor for the combined events during our long-term follow-up course. Then, the survival ROC curve was used and the optimal cutoff level of cellular or fibrocellular crescents was 7.39%. The survival from a combined event declined up to greater than or equal to 7.39% of glomeruli with crescents in the total cohort. Using a multivariate model, crescent in greater than or equal to 7.39% of glomeruli was confirmed as an independent predictor of the composite events with a higher hazard ratio. Nevertheless, the predictive value of crescents in less than 7.39% of glomeruli was not statistically significant. Considering most of patients in our cohort receiving immunosuppressive therapy, it might be attributed to that crescents in a minority of glomeruli representing a lesion reversible. The predictive value of crescents in greater than or equal to 7.39% was also validated in the sub-group analyses.

This retrospective and observational study has the following limitations: (1) It should be noted that our article only applied to well defined cellular or fibrocellular crescents, other than fibrous crescents, and the latter being composed of more than 75% fibrous matrix and less than 25% cells and fibrin [6]. As there was an excellent interobserver agreement among pathologists in identifying the fraction of glomeruli showing cellular plus fibrocellular crescents in the original Oxford study, this was not the case with fibrous crescents, which may be difficult to distinguish from sclerosis of the glomerular tuft with an associated capsular adhesion [15]. (2) The interobserver and intraobserver reproducibility of the pathologic diagnosis are needed. (3) The study population was from a single center and the ROC analysis might be weak, and the larger and multi-center cohort study is needed in the future.

In conclusion, we proposed that the crescents were not uncommon with important clinical significance, and the cutoff point of crescents as prognosticator might be nearly 7.39% in lupus nephritis based on our well-defined cohort. Including the extracapillary proliferation in the 2018 ISN/RPS revision system could improve more values for supporting clinical strategy.

## Supplementary Material

Supplemental Material Figure 1

Supplemental Material Table 1
