# Supplementary material for: The predictive value of crescents in the disease progression of lupus nephritis based on the 2018 International Society of Nephrology/Renal Pathology Society Revision System: a large cohort study from China
Source: Ren Fail. 2020 Feb 13;42(1):166–72. doi: 10.1080/0886022X.2020.1726385 (PMC7034106; doi:10.1080/0886022X.2020.1726385)
Supplement: Supplemental Material Figure 1 [file IRNF_A_1726385_SM7269.docx]

| **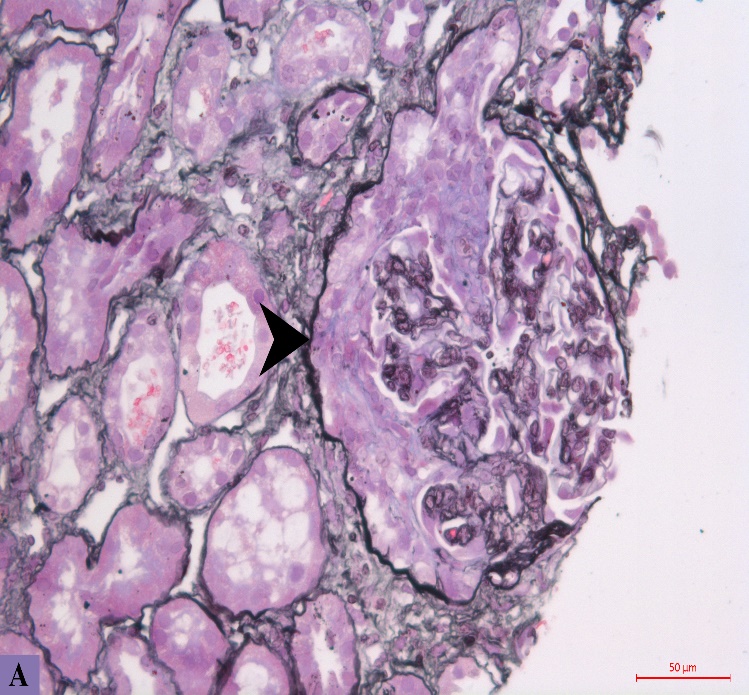** | **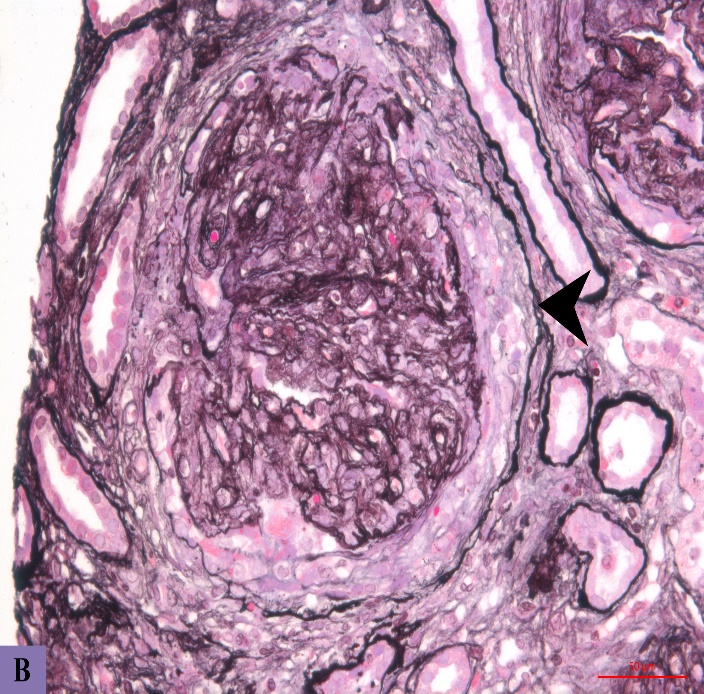** |
| --- | --- |

**Supplementary Figure 1.** **Crescent lesions for which recommendations were made in the text.** Arrows point to typical examples. Periodic Acid-Silver Methenamine and Masson´s trichrome staining; original magnification×400. (A) cellular crescent; (B) fibrocellular crescent.


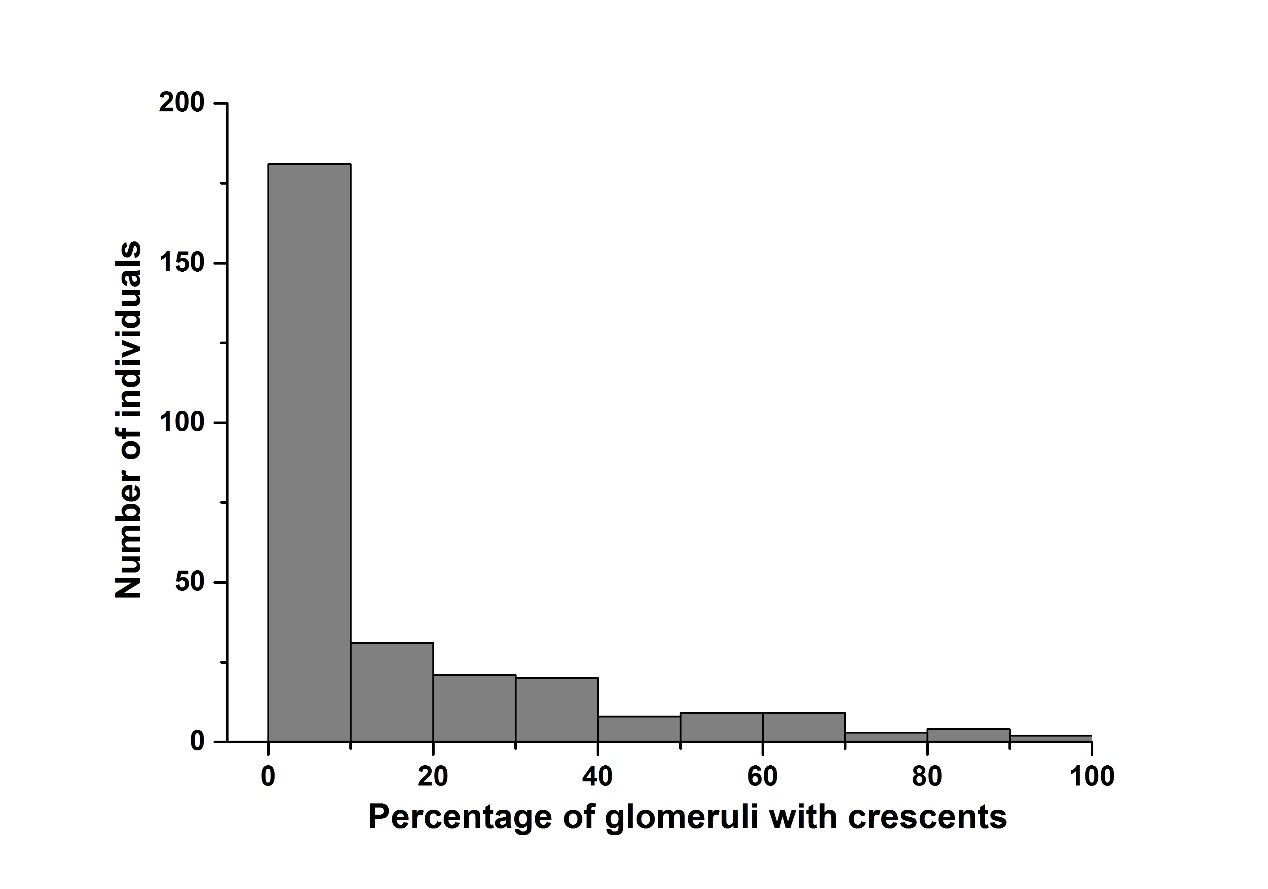


**Supplementary Figure 2．Distribution of the percentage of glomeruli with crescents in lupus nephritis.**

**Supplementary Figure 3．Kaplan-Meier analysis of the correlations between 7.39% of glomeruli with crescents and death in all patients**
