# Supplementary material for: The predictive value of crescents in the disease progression of lupus nephritis based on the 2018 International Society of Nephrology/Renal Pathology Society Revision System: a large cohort study from China
Source: Ren Fail. 2020 Feb 13;42(1):166–72. doi: 10.1080/0886022X.2020.1726385 (PMC7034106; doi:10.1080/0886022X.2020.1726385)
Supplement: Supplemental Material Table 1 [file IRNF_A_1726385_SM7246.docx]

**Supplementary Table 1. Association between crescents and clinicopathological characteristics at the time of renal biopsy**

| **The percentage of crescents** | | | | | | | |
| --- | --- | --- | --- | --- | --- | --- | --- |
| **Clinical data** | |  | ***P* Value** | **Pathological data (light microscopy)** | |  | ***P* Value** |
| **Gender** | **Female** | **17.9% (0, 21.7%)** | **0.5** | **Endocapillary hypercellularity** | **Yes** | **9.5% (0, 29.3%)** | **<0.001** |
|  | **Male** | **21.7% (0, 28.7%)** |  |  | **No** | **0 (0, 0)** |  |
| **^*^Hypertension** | **Yes** | **10.0% (0, 33.3%)** | **<0.001** | **Neutrophils/karyorrhexis** | **Yes** | **12.5% (0, 32.7%)** | **<0.001** |
|  | **No** | **0 (0, 6.3%)** |  |  | **No** | **0 (0, 6.3%)** |  |
| **Neurological disorder** | **Yes** | **14.9% (0, 21.1%)** | **0.2** | **Hyaline deposits** | **Yes** | **8.0% (0, 24.9%)** | **0.007** |
|  | **No** | **14.3% (0, 31.9%)** |  |  | **No** | **0 (0, 17.9%)** |  |
| **Anemia** | **Yes** | **8.2% (0, 32.1%)** | **<0.001** | **Interstitial inflammation** | **<25%** | **0 (0, 0)** | **<0.001** |
|  | **No** | **0 (0, 9.2%)** |  |  | **25-50%** | **0 (0, 16.7%)** |  |
| **Leukocytopenia** | **Yes** | **0 (0, 16.0%)** | **0.5** |  | **>50%** | **31.9% (6.2%, 56.0%)** |  |
|  | **No** | **2.3% (0, 22.7%)** |  | **Interstitial fibrosis** | **<25%** | **0 (0, 17.9%)** | **<0.001** |
| **Thrombocytopenia** | **Yes** | **0.9% (0, 24.0%)** | **0.9** |  | **25-50%** | **0 (0, 14.1%)** |  |
|  | **No** | **2.2% (0, 21.3%)** |  |  | **>50%** | **30.9% (4.7%, 55.8%)** |  |
| **Hematuria** | **Yes** | **5.9% (0, 26.7%)** | **<0.001** | **Tubular atrophy** | **<25%** | **0 (0, 14.8%)** | **<0.001** |
|  | **No** | **0 (0, 0)** |  |  | **25-50%** | **0 (0, 17.9%)** |  |
| **Leukocyturia** | **Yes** | **9.3% (0, 30.8%)** | **<0.001** |  | **>50%** | **34.7% (8.0%, 58.2%)** |  |
|  | **No** | **0 (0, 6.3%)** |  |  | | **r Value** | ***P* Value** |
| **Nephrotic syndrome** | **Yes** | **4.8% (0, 27.1%)** | **0.007** | **AI** | | **0.671** | **<0.001** |
|  | **No** | **0 (0, 12.8%)** |  | **Fibrinoid necrosis** | | **0.258** | **<0.001** |
| **Acute kidney injury** | **Yes** | **31.0% (6.5%, 50.8%)** | **<0.001** | **CI** | | **0.265** | **<0.001** |
|  | **No** | **0 (0, 10.8%)** |  | **Global and/or segmental sclerosis** | | **0.068** | **0.3** |
| **Positive ANA** | **Yes** | **2.2% (0, 22.0%)** | **0.3** | **Fibrous crescents** | | **0.139** | **0.02** |
|  | **No** | **0 (0, 12.1%)** |  | **Pathological data (direct immunofluorescence)** | | **r Value** | ***P* Value** |
| **Positive anti-ds-DNA** | **Yes** | **6.0% (0, 26.8%)** | **0.001** | **IgG** | | **-0.250** | **<0.001** |
|  | **No** | **0 (0, 10.3%)** |  | **IgA** | | **-0.059** | **0.3** |
|  | | **r Value** | ***P* Value** | **IgM** | | **-0.096** | **0.1** |
| **Serum creatinine** | | **0.473** | **<0.001** | **C3** | | **0.041** | **0.5** |
| **Proteinuria** | | **0.160** | **0.006** | **C1q** | | **0.087** | **0.1** |
| **Age** | | **0.083** | **0.2** | **Fibrin** | | **0.061** | **0.3** |
| **C3** | | **-0.168** | **0.004** |  | |  |  |
| **SLEDAI** | | **0.274** | **<0.001** |  | |  |  |
| **Hemoglobin** | | **-0.290** | **<0.001** |  | |  |  |

**Notes: ^*^Hypertension, blood pressure≥140/90mmHg; SLEDAI, systemic lupus erythematosus disease activity index; ANA, anti-nuclear antibodies; dsDNA, double-stranded DNA; AI, NIH activity index; CI, NIH chronicity index. Non-parametric variables were expressed as median (interquartile range) and compared using either Mann-Whitney or Kruskal-Wallis.**
